# Fast volume-scanning light sheet microscopy reveals transient neuronal events

**DOI:** 10.1364/BOE.9.002154

**Published:** 2018-04-09

**Authors:** Peter Haslehurst, Zhengyi Yang, Kishan Dholakia, Nigel Emptage

**Affiliations:** 1 Department of Pharmacology, University of Oxford, Oxford, OX1 3QT, UK; 2 SUPA, School of Physics & Astronomy, University of St Andrews, St Andrews, KY16 9SS, UK; 3 these authors contributed equally to this work; 4 kd1@st-andrews.ac.uk; 5 nigel.emptage@pharm.ox.ac.uk

**Keywords:** (170.0180) Microscopy, (170.3880) Medical and biological imaging, (180.2520) Fluorescence microscopy, (170.2655) Functional monitoring and imaging

## Abstract

Light sheet fluorescence microscopy offers considerable potential to the cellular neuroscience community as it makes it possible to image extensive areas of neuronal structures, such as axons or dendrites, with a low light budget, thereby minimizing phototoxicity. However, the shallow depth of a light sheet, which is critical for achieving high contrast, well resolved images, adds a significant challenge if fast functional imaging is also required, as multiple images need to be collected across several image planes. Consequently, fast functional imaging of neurons is typically restricted to a small tissue volume where part of the neuronal structure lies within the plane of a single image. Here we describe a method by which fast functional imaging can be achieved across a much larger tissue volume; a custom-built light sheet microscope is presented that includes a synchronized galvo mirror and electrically tunable lens, enabling high speed acquisition of images across a configurable depth. We assess the utility of this technique by acquiring fast functional Ca^2+^ imaging data across a neuron’s dendritic arbour in mammalian brain tissue.

## 1. Introduction

The neuron is a sophisticated computing device, operating on millisecond time scales to perform complex integrating functions that are not yet fully understood. A principle neuron of the mammalian cortex or hippocampus has an elaborate arbour of long (hundreds of micrometre) processes known as dendrites which are typically covered in tens of thousands of sub-micrometre structures known as spines. Each spine represents the post-synaptic half of a synapse – the intricate structure which mediates the transmission of incoming signals to the neuron. Synaptic transmission causes rapid post-synaptic events such as depolarization of the membrane potential and localized influx of ions such as Ca^2+^ and Na^+^, which neuroscientists can detect using an array of inorganic or genetically-encoded dyes whose fluorescence varies with biologically significant parameters such as voltage or ionic concentrations. These dyes can be introduced into the neuron by viral transfection or patch pipette. Classically these dyes have been used with a confocal microscope configured to make a linear scan across a single spine. Fast 2D scanning of a larger area would allow transmission events to be monitored over several dendrites and multiple spines, but 2D confocal scanning is too slow to achieve the 10 or 20 ms scan times required to trace these events over anything but the smallest area. This limitation has two important consequences: 1) Rapid transmission events can be traced only over a very limited set of structures, typically a single spine and the immediately adjacent dendrite. 2) The decision about which spine is of interest must be made before image acquisition begins – interesting events may be occurring at other spines or elsewhere in the dendritic arbour, but will not be captured by the experiment.

Cellular neuroscientists are beginning to turn to light sheet fluorescence microscopy (LSFM) to overcome these limitations. Instead of scanning with a focussed single point of light as in confocal microscopy, LSFM typically uses a cylindrical lens to focus a collimated laser beam in one plane, which when directed through the illumination objective produces a thin sheet of light rather than a point. The detection objective is arranged at right angles to the illumination objective so that its focal plane coincides with the plane of the light sheet. A fast sCMOS (scientific complementary metal oxide semiconductor) camera enables rapid acquisition of an image from the whole field of view [1]. LSFM was pioneered in the field of developmental biology, where a specimen such as a fruit fly or zebrafish embryo is embedded in agarose gel and positioned in the plane of the light sheet, often being rotated during imaging to acquire an image stack that includes the whole organism [2]. In such a setup the two objective lenses are usually arranged in the horizontal plane [3]. Recently we described a modification of this setup which places two water-immersion objective lenses in the vertical plane pointing downwards into a custom-built perfusion chamber [4]. This geometry was driven by a desire to harness the potential of LSFM for imaging rapid events in living neurons, so the design was configured to allow mammalian brain slices (such as cultured slice preparations from rat hippocampus) to be perfused with oxygenated artificial cerebro-spinal fluid (ACSF) and hence maintained in healthy condition throughout the imaging session.

In using this setup we encountered a key limitation – to capture rapid post-synaptic events such as transient Ca^2+^ influx it is necessary to acquire with a temporal resolution of 50-100 volumes/sec. This restricts us to imaging in single-plane mode (XYT), because acquisition of an XYZT stack requires physical movement of the sample stage by a piezoelectric motor, which dramatically slows down the acquisition rate. If we accept this restriction and image in single-plane mode, we find that the meandering path traced by dendrites through a brain slice means that we can only image those short lengths of the dendrite which happen to coincide with the plane of the light sheet (Fig. 4). The present study addresses this key shortcoming and presents a design which enables fast volume imaging without physical movement of the sample.

Several different approaches to fast volume imaging with LSFM have already been reported, most of which involve scanning the light sheet up and down the detection axis by means of a scanning mirror. To achieve in-focus images, the shallow depth of field (DOF) of the detection objective must coincide with the light sheet. One approach to achieve this is to synchronize piezo-driven movement of the detection objective with movement of the light sheet to allow volume imaging without movement of the sample [5–7]. However the mass of the objective limits the scanning frequency to less than 10 volumes/sec. Another strategy has been to extend the DOF of the detection objective so the sample is always in focus [8–11]. This can be achieved by using a dynamic [8] or static [9] cubic phase mask, harnessing spherical aberration [10], or rapidly scanning the detection plane with a tunable acoustic gradient lens (TAG lens) [11]. However, because of the extended DOF the point spread function along the detection axis is elongated, compromising optical efficiency and signal-to-noise ratio. Single objective scanning methods such as oblique-plane microscopy (OPM) [12] and swept confocally-aligned planar excitation (SCAPE) microscopy [13] are able to image at rates up to 20 volumes/sec, but demand complex optical arrangements.

Another approach is to introduce an electrically tunable lens (ETL) into the detection arm of the microscope, synchronizing movement of the imaging plane with the scanning movement of the light sheet, so that the image formed on the camera's detector is always in focus [14]. This has achieved some success imaging embryonic chick or zebrafish beating heart [14,15] with acquisition speeds up to 30 volumes/sec. This frame rate was only achieved by substantially cropping the field of view. For our purposes – the visualization of rapid calcium signalling events in the dendrites of a living neuron – the structure of the neuron is static while the signal intensity changes rapidly. Hence only one projection image is required per volume to resolve fast calcium events. An image stack for the purpose of recording the 3D structure of the neuron need only be acquired once, at the beginning or end of the experiment.

**Fig. 1:**
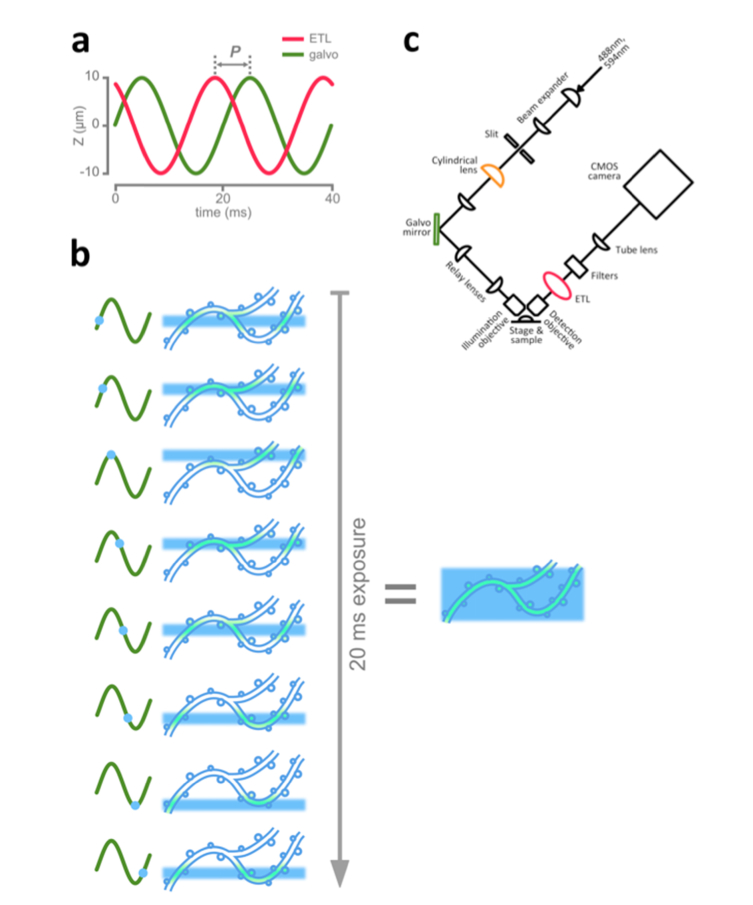
Inverted light sheet microscope modified for fast volume scanning. a: The control signals applied to the electrically tuneable lens (ETL) and the galvo mirror. These move the light sheet and the focal plane of the detection objective in synchrony through a 20 µm traverse in the Z axis. In practice the control signals need to be separated by a phase difference (P) because of the lag time required for the ETL to respond to the signal. b: Diagram illustrating how fast volume scanning builds up a deeper image volume. In the left panel is shown the position of the light sheet illuminating a neuronal dendrite at consecutive moments as it scans through a complete cycle covering 20 µm in the Z axis. The right panel shows the effective image depth acquired over the course of a 20 ms camera exposure. c: Schematic diagram of the modified inverted LSM described in this paper.

We implement this by synchronizing the movements of the detection plane and the light sheet, while setting the camera's exposure time to equal the period of the scanning cycle (Fig. 1(b)

). This allows us to use the camera's full frame, and dramatically reduces the amount of data generated. Specifically, we position a 1D galvo mirror in the illumination light path, and an ETL behind the detection objective (Fig. 1(c)). The galvo mirror and the ETL are each driven by a 50 Hz sine wave electrical signal. These two signals are configured with a phase shift to allow for the delay between a change in the driving signal and the corresponding physical change in the ETL’s curvature (Fig. 1(a)). Thus, during the course of the camera’s 20 ms exposure time the light sheet and the detection objective’s focal plane both make one complete scan through the detection (Z) axis. The depth of this scan can be adjusted by altering the amplitudes of the driving signals; in our experiment we scanned through a depth of 20 μm. Effectively each 20 ms exposure collects an average intensity projection of an XYZ scan, and the resulting image file is equivalent to an XYT stack collected with an effective light sheet thickness of 20 μm. This fast volume-scanning LSFM approach allows us to image much longer segments of dendrite than is feasible with single-plane LSFM, as we track fast Ca^2+^ events with high temporal resolution. Here we present data from an example experiment which illustrate the promise of fast volume-scanning LSFM for cellular neuroscience.

## 2. Methods

### Optical setup

The optical setup is based on the inverted light sheet microscope (LSM) previously reported [4]. Briefly, a 488 nm wavelength laser (STRADUS-488-150, 150 mW, Vortran) and a 594 nm wavelength laser (31-2230, Coherent) are coupled into a single mode fibre which guides the beam into the imaging setup. The illumination beam is collimated by a fibre collimator (CFC-11X-A, Thorlabs), then expanded by a beam expander. A cylindrical lens (CL, LJ1695RM-A, FL 50 mm, Thorlabs) focuses the beam to a light sheet. After passing through a relay lens combination and the illumination objective (UMPLFLN 10XW NA 0.3, water dipping, Olympus), the light sheet is formed in the sample chamber. The image is collected by the detection objective (LUMPLFLN 40XW NA 0.8, water dipping, Olympus), then focussed by an achromatic tube lens (LA1708-A-ML, FL 200 mm, Thorlabs) onto the sCMOS camera (ORCA-Flash4.0, Hamamatsu). An automatic stage (M-110.12S, PI) can be used to scan the sample chamber along the detection axis to build up a complete 3D image stack.

Fast volume scanning is achieved by scanning the illumination light sheet axially with a galvo mirror (GVS001, Thorlabs) on the illumination path. At the same time, an ETL (EL-10-30-C, Optotune) located immediately behind the detection objective scans the detection plane. Although it is recommended to position the ETL horizontally to minimize optical aberration, we found that placing the ETL at 45 degrees to horizontal has a negligible effect on image quality. Previous studies [14,15] utilized a 4f system to relay the back aperture of the imaging objective to the ETL to minimise the influence on magnification. In our compact arrangement, because of the small axial scanning range, the magnification change is very limited [16].

The motions of the detection plane and the illumination light sheet are synchronized so that they always coincide. The trigger output signal from the ETL driver is passed to a function generator (LXI3390, Keithley) which controls the timing of the scanning galvo mirror. The function generator is operated in burst mode to periodically reset the hardware synchronization between the galvo mirror and the ETL.

### Brain slice preparation

Experiments were conducted in accordance with current United Kingdom (UK) legislation regulating the welfare of animals used for scientific procedures. Organotypic slices were prepared as previously described [4,17]. In summary, transverse sections of hippocampus (thickness 350 μm) were prepared from male Wistar rat pups (postnatal day 7, Envigo) and incubated (34.5 °C, 5% CO_2_) on Millicell culture inserts (Millipore) in culture medium for 2 weeks before use. The culture medium comprised 79% Minimum Essential Media, 20% heat-inactivated horse serum, 1% B-27 (Invitrogen), 30 mM HEPES, 26 mM glucose, 5.8 mM NaHCO_3_, 1 mM CaCl_2_, and 2 mM MgSO_4_. Culture medium was replaced twice weekly. A pyramidal neuron in the CA3 region of the hippocampus was briefly filled with synthetic dyes AF594 (0.5 mM, Life Technologies) and OGB-1 (0.5 mM, Life Technologies) via a patch pipette. After filling, the organotypic slice was transferred to the LSM’s recording chamber (capacity ~4 ml) and perfused at 2 ml/min with carbogenated (95% O_2_/5% CO_2_) ACSF. The composition of this ACSF was: (mM) 145 NaCl, 16 NaHCO_3_, 11 glucose, 2.5 KCl, 1.2 NaH_2_PO_4_, 3 CaCl_2_, and 1 MgCl_2_, supplemented with 1 mM Trolox to minimize phototoxic damage during imaging. In order to stimulate spiking activity in the slice, about 1 min before imaging the perfusion pump was stopped and a small amount (500 μl) of high-K^+^ ACSF was added to the perfusion chamber. The composition of this high-K^+^ ACSF was: (mM) 107.5 NaCl, 40 KCl, 16 NaHCO_3_, 11 glucose, 1.2 NaH_2_PO_4_, 3 CaCl_2_, and 1 MgCl_2_.

### Imaging calibration

Prior to the experiment, on the same day, the fast volume-scanning LSM was configured by imaging 500 nm fluorescent beads (Tetraspeck) suspended in agarose. The ETL and galvo mirror were configured to sweep through approximately 20 μm in the Z axis (i.e. axial with respect to the detection objective) every 20 ms. The phase difference between the ETL control signal and the galvo mirror control signal was adjusted to optimize image quality (Fig. 1(a)).

### Data analysis

Initial image analysis was done using Fiji [18]. A custom-written macro was used to select regions of interest (ROIs) at various locations on the dendritic tree, along with a larger nearby ROI to represent background fluorescence, and extract mean intensity data for each time point. R scripts [19] were custom written to plot the intensity traces (calculated as ΔF/F after subtraction of an exponential decay function fitted to background) and to fit a rise/decay function (a + b.t.e^-c.t^ where a, b, and c are constants and t is time) [20] to candidate Ca^2+^ events. Candidate events were only accepted if the amplitude of the event (calculated from the fitted rise/decay function) was greater than the estimated noise (calculated as the root mean square of the baseline intensity trace for the 1 second directly preceding the candidate event (see Fig. 6 in the Appendix for details). The time course of the Ca^2+^ events detected using volume scanning LSFM was comparable with that of Ca^2+^ events detected using single-plane LSFM (see Table 1 in the Appendix).

## 3. Results

To demonstrate the potential of our approach, we present data acquired from a single imaging experiment, where the dendritic arbour of a living pyramidal neuron was scanned for 10 seconds using fast volume-scanning LSFM. *Post hoc* analysis of the image data reveals details of localized, rapid Ca^2+^ influx events occurring at various locations and their spread or otherwise through the dendritic arbour. This wealth of information cannot be acquired by a classical confocal line scan approach, which by its nature requires the investigator to decide ahead of time upon a single location of interest.

**Fig. 2:**
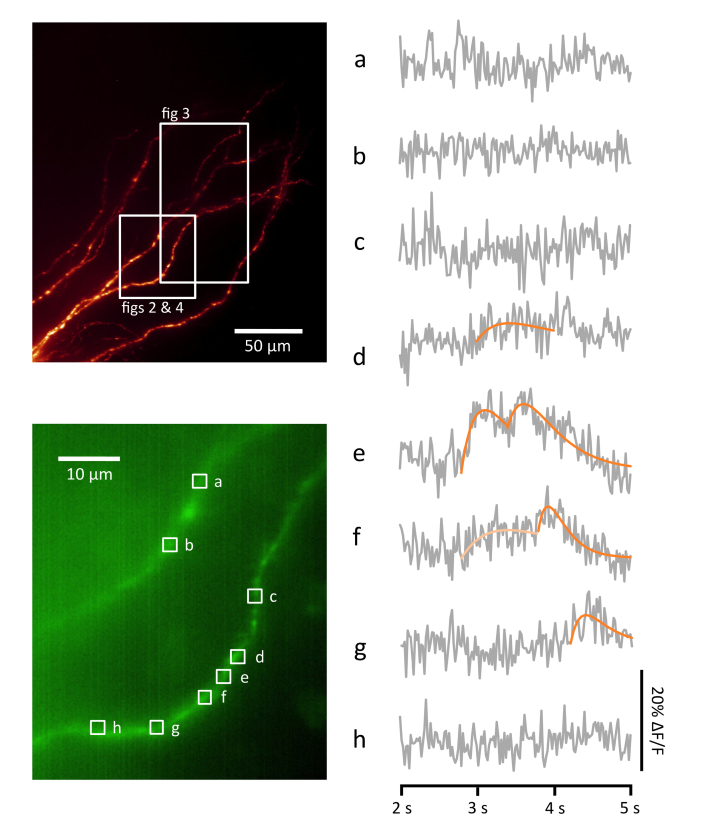
Example Ca^2+^ events detected using fast volume-scanning LSM. Upper left panel: maximum intensity projection of a portion of the basal dendritic arbour of a pyramidal neuron in a rat hippocampal brain slice. The neuron was filled with Alexa Fluor 594 (AF594) and Ca^2+^-sensitive dye Oregon Green BAPTA-1 (OGB-1), and imaged with 594 nm illumination. The volume shown in this image has a depth of 160 µm. Lower left panel: average intensity projection of a fast volume scan acquired from the same neuron using 488 nm illumination. The volume scanned has a depth of ~20 µm. The volume was imaged for a duration of 10 sec, with the whole volume being scanned once every 20 ms (50 volumes/sec). Right panel: shows changes in OGB-1 fluorescence at various regions of interest (a-h) during a 3 sec period of the scan starting at the 2 sec time point (grey). Ca^2+^ transient events are clearly visible in some but not all of the traces, and have been fitted with a rise/decay function (orange; see Methods). Note that the first candidate event in trace (f) did not pass the criteria to be identified as an event (light orange; see Fig. 6).

Using a patch electrode, we filled a pyramidal neuron in the CA3 region of a cultured rat hippocampal slice with Ca^2+^-sensitive dye Oregon Green BAPTA-1 (OGB-1), along with Alexa Fluor 594 (AF594) for structural imaging, then transferred the slice to the LSM. About 1 minute before the start of image acquisition, a small amount of high-K^+^ ACSF was added to the recording chamber to stimulate spiking activity in the hippocampal slice. A portion of the filled neuron’s basal dendritic arbour was imaged using the fast volume-scanning technique described above (Fig. 1). The image acquisition ran for 10 seconds with an exposure time of 20 ms (50 volumes/sec). In Fig. 2

we show the changes in OGB-1 fluorescence at various locations in the dendritic arbour, using data taken from the segment of the recording that was acquired between the 2 sec and 5 sec time points. Where transient events are apparent, a rise/decay function has been fitted (orange lines). Notice that a strong double-peaked Ca^2+^ transient event is observed at region of interest (ROI) e at about the 2.8 sec time point. A short distance (less than 5 μm) along the dendrite in either direction (ROIs f and d) the same event is apparent but with a slower rise time and a much reduced amplitude. Further (about 10 μm) towards the soma the first event is not visible, while the second event is still visible though reduced in amplitude and delayed (ROI g). Further away from ROI e (ROIs c and h) or in another dendrite (ROIs a and b) there is no sign of the event, showing that this cannot be interpreted as a back-propagating action potential originating from the soma, as this would be visible throughout the dendritic arbour. It is likely that this segment of the recording shows a strong synaptic transmission event (or sequence of two events) occurring at one or two synapses in the vicinity of ROI e. The resulting Ca^2+^ influx is seen propagating a short distance along the dendrite on either side of the event’s origin, possibly reflecting calcium-induced calcium release (CICR) [21], or even a regenerative dendritic spike [22], but it is noteworthy that the propagating event decays away within a short distance (about 10 μm) of its probable origin.

In Fig. 3

**Fig. 3:**
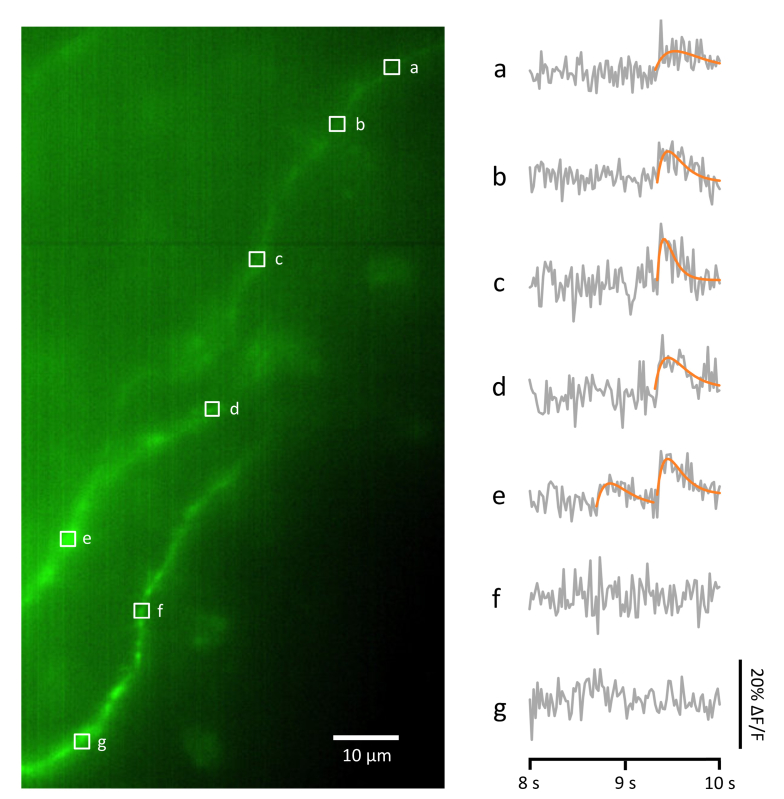
Further example Ca^2+^ events detected using fast volume-scanning LSM. This figure shows other Ca^2+^ events taken from the same 10 sec fast volume scan that is shown in Fig. 2. Left panel: average intensity projection of a fast volume scan acquired using 488 nm illumination, showing a small segment of the dendritic arbour of the same pyramidal neuron depicted in Fig. 2, filled with OGB-1. The volume scanned was ~20 µm deep. Right hand panel: changes in OGB-1 fluorescence at various regions of interest (a-g), during the final 2 sec of the 10 sec scan (grey). Ca^2+^ transient events are clearly visible in some but not all of the traces, and have been fitted with a rise/decay function (orange; see Methods).

we show the same analysis applied to a different time segment taken from the same recording, between the 8 sec and 10 sec time points. Here we see a Ca^2+^ transient event starting at about the 9.3 sec time point, which seems to have the sharpest rise time and largest amplitude at ROI c, but is also visible at least 50 μm away in either direction along the dendrite (ROIs a,b,d and e), but not on a different dendrite (ROIs f and g), which again rules out a back-propagating action potential as an interpretation. Note that at ROI e there is another, earlier Ca^2+^ transient event at about the 8.6 sec time point which in contrast does not propagate very far along the dendrite. A likely interpretation is that we have two synaptic transmission events: one at a synapse in the vicinity of ROI c which engages some kind of amplifying mechanism such as CICR or dendritic spiking to enable the Ca^2+^ influx to propagate at least 50 μm along the dendrite; and another transmission event at a synapse in the vicinity of ROI e which fails to propagate a Ca^2+^ influx along the dendrite.

**Fig. 4:**
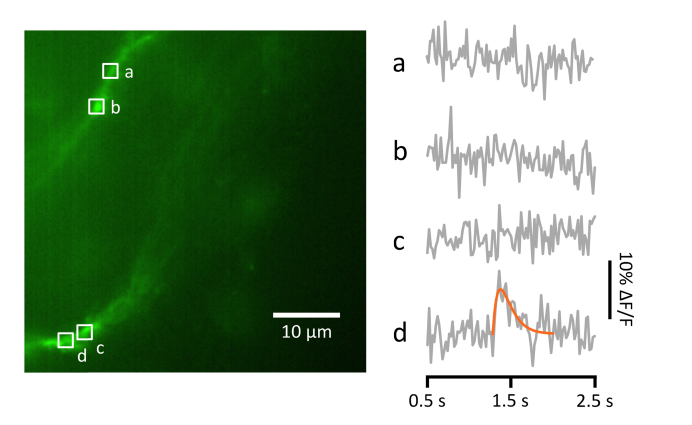
Example Ca^2+^ event detected using single-plane LSFM. Left panel: average intensity projection of a single-plane LSFM scan acquired from the same neuron as shown in Fig. 2 and Fig. 3 using 488 nm illumination. The image was acquired over 10 sec, with an exposure time of 20 ms (50 volumes/sec). Compare with Fig. 2 – here only short lengths of dendrite are in focus. Right panel: changes in OGB-1 fluorescence at various regions of interest (a-d) during a 2 sec period of the scan starting at the 1.5 sec time point (grey). A Ca^2+^ transient event is apparent in one of the traces, and has been fitted with a rise/decay function (orange; see Methods).

For comparison a second 10 sec image acquisition was performed on the same neuron, but this time the LSM was operated in single-plane mode (Fig. 4

). As before spiking was encouraged by addition of a small amount of high-K^+^ ACSF. Comparison with Fig. 2 shows that the single-mode image stack has less of the dendritic tree in focus. This restricts the usefulness of the analysis compared with fast volume-scanning mode, as only short stretches of dendrite are enough in focus to yield a meaningful trace. In Fig. 4 we show OGB-1 fluorescence changes between the 0.5 sec and 2.5 sec time points at various ROI’s on the limited stretches of dendrite that are in focus. A single Ca^2+^ transient event is visible at about the 1.3 sec time point at ROI d. This event does not appear to propagate far along the dendrite, but it is hard to make a firm interpretation because of the fragmentary nature of the in-focus dendrites available in this single-plane image stack.

**Fig. 5:**
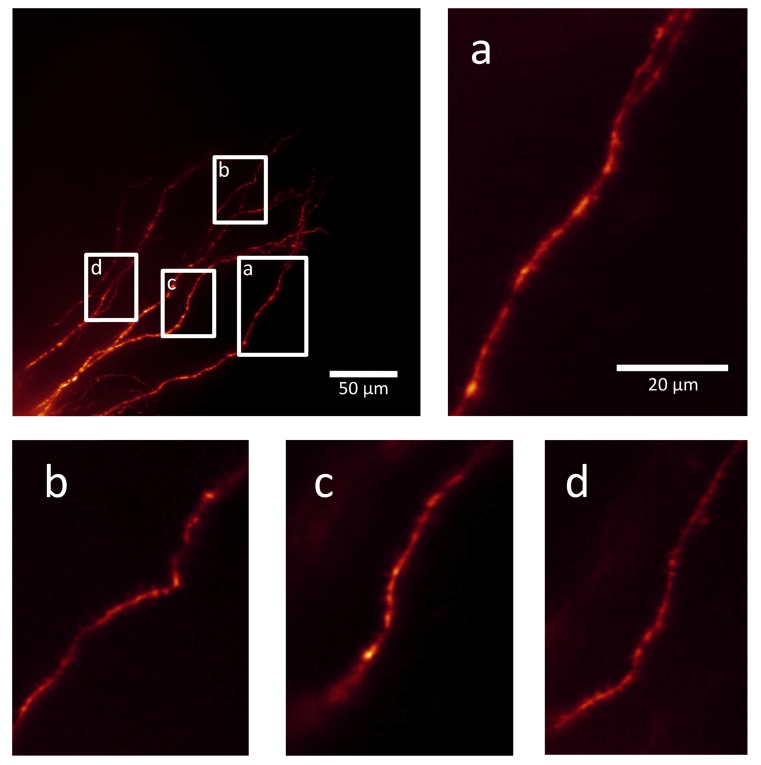
Our fast volume-scanning LSM is also capable of acquiring high resolution structural images. Upper left panel: maximum intensity projection of a portion of the basal dendritic arbour of the same hippocampal pyramidal neuron shown in Figs. 2-4. The neuron was filled with AF594 and imaged with 594 nm illumination and 200 ms exposure time. The image stack was built up of 320 image steps at intervals of 0.5 µm, hence the image has a depth in the Z axis of 160 µm. Panels a, b, c and d are details of various parts of this field of view, cropped from the same image stack. The 20 µm scale bar in panel a applies to all 4 of these panels. Panel a is a single image slice, panels b, c and d are maximum intensity projections of 4, 4, and 7 contiguous slices respectively.

**Fig. 6:**
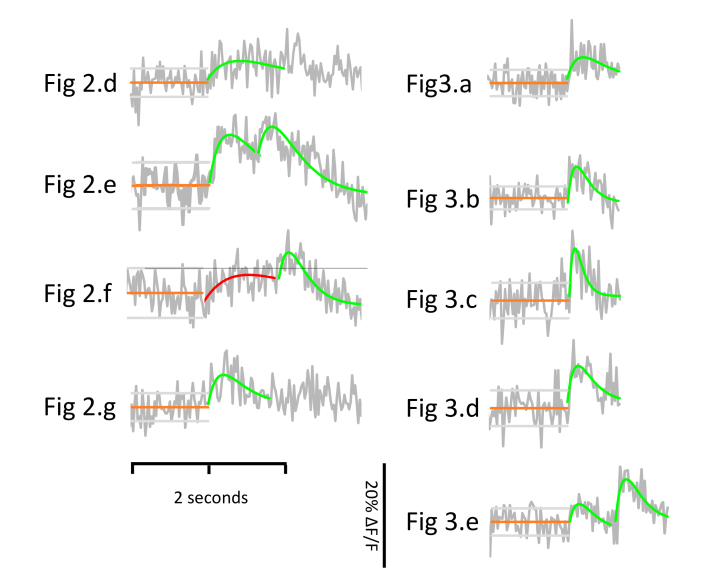
Illustration of criteria used to identify Ca^2+^ events. The events shown in Fig. 2 and Fig. 3 are plotted again along with the preceding 1 second baseline period. The mean baseline is shown (orange) along with an estimate of noise (grey) calculated as ± root mean square (RMS) of the baseline signal. Candidate events were fitted with a rise/decay function (see Methods). Each candidate event was accepted (green) if its amplitude exceeded mean baseline + RMS. One example is shown of a candidate event which narrowly missed being identified as an event (red).

**Fig. 7:**
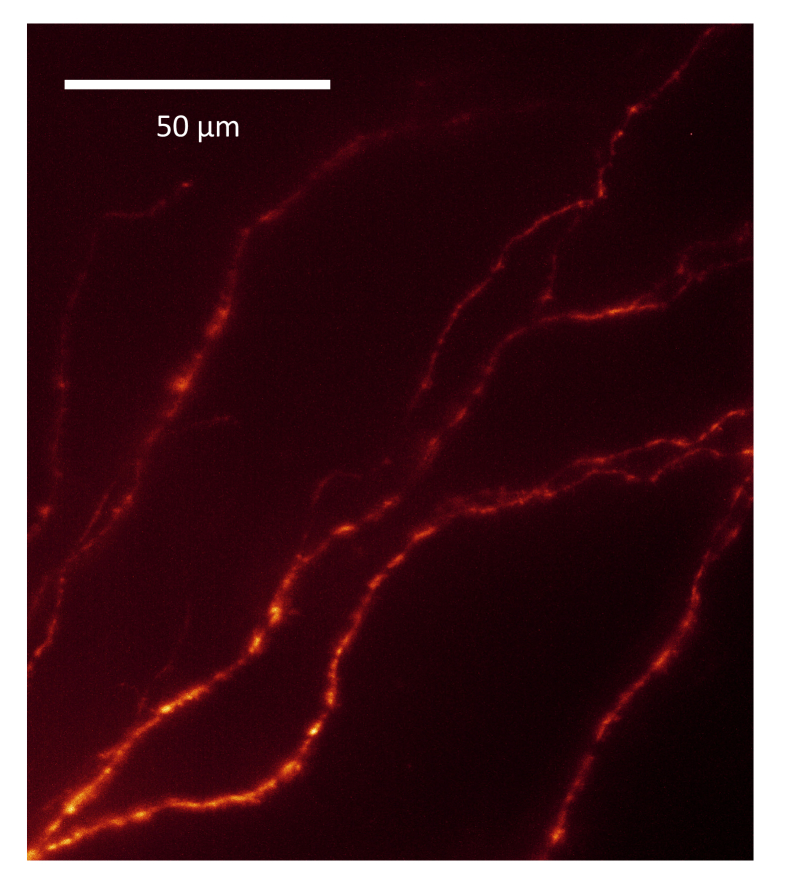
Sectioning properties of the LSM in the Z-axis. Maximum intensity projection of a region of interest of the same neuron shown in previous figures. The neuron was imaged by the LSM in single-plane mode with 594 nm illumination, 200 ms exposure, and 1 µm steps in the Z-axis between each image in the stack. The associated video steps through this image stack at 4 frames per second (see Visualization 1).

We also show details of a structural image stack acquired at the close of the same experiment (Fig. 5

). Using 594 nm illumination and an exposure time of 200 ms, a large image stack was acquired using the conventional method of incrementally moving the stage by 0.5 µm in the Z axis between each exposure. (See also Fig. 7 in the Appendix and Visualization 1.) This demonstrates that our custom-built fast volume-scanning LSM is also capable of acquiring structural images with spine-level resolution.

## 4. Discussion

In this report we have presented imaging data acquired by a variant of our previously reported neuroscience-optimized LSM [4] modified to enable fast scanning of the light sheet in the Z axis. We have shown that this approach enables simultaneous tracking of multiple transient Ca^2+^ influx events across several dendrites and is able to monitor longer contiguous sections of dendrite than single-plane LSFM. Other groups have described LSMs which, like our design, use a galvo mirror to move the light sheet rapidly in the Z axis, along with an ETL which moves the detection objective’s focal plane to track the movement of the light sheet [14,15]. However, to our knowledge, the design we describe here is the first report that specifically addresses the problem of obtaining XYT image stacks with high temporal resolution from long thin structures such as neuronal dendrites, the courses of which meander through the sample tissue (in this case a hippocampal brain slice) in all three dimensions. If the average intensity projection images obtained with fast volume-scanning LSFM (Fig. 2 and Fig. 3) are compared with that obtained with single-plane LSFM (Fig. 4), one can see that the single- plane image has slightly better resolution, with small objects such as spines being a little more visible. However, before jumping to the conclusion that single-plane LSFM is “better” than fast volume-scanning LSFM, there are two points to consider: 1) These image were acquired using Ca^2+^-sensitive dye OGB-1; this dye is designed to report rapid changes in intracellular Ca^2+^ concentration. In baseline conditions dyes such as OGB-1 are relatively dim. For structural imaging one would use a bright fluorescent dye such as AF594 whose brightness is not affected by intracellular conditions, and fast scanning techniques would be unnecessary. 2) Fast volume-scanning LSFM and single-plane LSFM are optimized for different tasks. Single-plane LSFM is best suited to an experiment which is interested in fast functional imaging in a restricted length of dendrite of the order of 20 μm long, where perhaps a single synapse might be manipulated using a technique such as glutamate uncaging, and the functional effect of this on a handful of nearby spines monitored with high temporal resolution. It is relatively simple to find such a short length of contiguous dendrite that is in the plane of the light sheet. Fast volume-scanning LSFM on the other hand is best suited to an experiment that is designed to collect fast functional imaging data over a wider area of the dendritic arbour, covering multiple dendrites and multiple spines in a single imaging scan. Note that the depth of the light sheet’s scan in the Z axis, which in our example experiment was configured at 20 μm, can be configured to any value consistent with the operational ranges of the signals driving the galvo mirror and the ETL.

We conclude that fast volume-scanning LSFM holds considerable promise for studies in cellular neuroscience. It has the potential to acquire fast functional imaging data across wide areas of a neuron’s dendrites. This raises the prospect of understanding in more detail than ever before the way that synaptic transmission events interact with events at neighbouring or more distant synapses, and are integrated and propagated (or suppressed) through various compartments and branches of the dendritic arbour.

## Appendix

**Table 1:** Time constants of Ca^2+^ events detected with volume-scanning or single-plane LSFM are comparable.

	Time constant (ms)		
	Volume scanning		Single plane
	421.9		97.1
	265.7		73.7
	256.1		82.7
	166.3		83.0
	200.5		101.0
	208.9		85.6
	114.4		359.7
	71.7		126.2
	140.0		72.9
	143.5		95.6
	141.2		

Mean	193.7		117.8
SE	28.8		27.3

Difference in means		75.9	
95%confidence interval		−2.0 to + 153.8	

Time constants of identified Ca^2+^ events were calculated from the fitted rise/decay curves. The volume-scanning data are taken from the experiments depicted in Figs. 2, 3 and 6. The single-plane data are taken from several experiments using single-plane LSFM including that depicted in Fig. 4. The 95% confidence interval for the difference between means of the two groups spans zero, showing that the time constants measured using the two different approaches are comparable.
